# The influence of disturbance scale on the natural recovery of biological soil crusts on the Colorado Plateau

**DOI:** 10.3389/fmicb.2023.1176760

**Published:** 2023-08-03

**Authors:** Sierra D. Jech, Caroline A. Havrilla, Nichole N. Barger

**Affiliations:** ^1^Department of Ecology and Evolutionary Biology, University of Colorado Boulder, Boulder, CO, United States; ^2^Department of Forest and Rangeland Stewardship, Colorado State University, Fort Collins, CO, United States

**Keywords:** biological soil crust, scale, natural recovery, Colorado Plateau, disturbance ecology, chlorophyll a, exopolysaccharides, soil stability

## Abstract

Up to 35% of global drylands have experienced degradation due to anthropogenic impacts, including physical disturbances like trampling and soil removal. These physical disturbances can result in the loss of soil communities known as biological soil crusts (biocrusts) and the important functions they provide, such as soil stability and fertility. The reestablishment of biocrust organisms after disturbance is determined by many factors, including propagule availability, climate, and vascular plant community structure. The role of these factors in natural recovery may be intensified by the extent (or size) of a disturbance. For example, large disturbances can result in reduced propagule availability or enhanced erosion, which impact both the dispersal and establishment of biocrust organisms on disturbed soils, leading to a slower natural recovery. To test how disturbance extent impacts biocrust's natural recovery, we installed four disturbance extents by completely removing biocrust from the mineral soil in plots ranging from 0.01 m^2^ to 1 m^2^ and measured productivity and erosion resistance. We found that small disturbance extents did not differ in chlorophyll a content, total exopolysaccharide content, or soil stability after 1.5 years of natural recovery. However, the concentration of glycocalyx exopolysaccharide was higher in the smallest disturbances after the recovery period. Our results indicate that disturbances <1 m^2^ in scale recover at similar rates, with soil stability returning to high levels in just a few years after severe disturbance. Our findings align with prior work on biocrust natural recovery in drylands and highlight the opportunity for future work to address (1) cyanobacteria, moss, and lichen propagule dispersal; (2) rates and mechanisms of biocrust succession; and (3) the role of wind or water in determining biocrust colonization patterns as compared to lateral growth.

## 1. Introduction

Biological soil crusts (biocrusts) are communities of moss, lichen, and cyanobacteria autotrophs as well as other microbial heterotrophs that live at the soil surface as a coherent layer or crust (Weber et al., 2022). Biocrusts can be a dominant biotic cover type in drylands (Rodriguez-Caballero et al., 2018), which make up ~40% of the global land area (Safriel et al., 2005). Biocrusts influence soil fertility and stability and interact with co-occurring plant communities (Mazor et al., 1996; Kohler et al., 2017; Havrilla et al., 2019). However, biocrust organisms are vulnerable to physical disturbance (e.g., trampling), with natural recovery being a relatively slow process (3–300 years; Table 23.1 in Weber et al., 2016).

The rate and trajectory of biocrust natural recovery, defined as successional patterns of recolonization (Weber et al., 2016), are controlled by a variety of interacting biotic and physical site factors, including climate, site stability, and disturbance history (Belnap and Eldridge, 2001). The combination of these factors makes it challenging to compare and predict the timeline and trajectory of biocrust's natural recovery following disturbance. For biocrust colonization to occur, biocrust propagules must disperse to the site (Bowker et al., 2010; Warren et al., 2018), attach to the surface, and then grow and reproduce. Propagule dispersal may be controlled by processes such as wind patterns (Dvorák et al., 2012; Barberán et al., 2015; Warren et al., 2018), overland flow, and lateral growth from existing biocrusts (Sorochkina et al., 2018; Yang et al., 2022). Propagule dispersal is considered a primary barrier to natural recovery (Bowker, 2007). Propagule attachment, growth, and reproduction may be dependent on local conditions, including soil texture (Chock et al., 2019; Faist et al., 2020), the presence of early successional species that facilitate the growth of other organisms (Read et al., 2016; Elliott et al., 2019; Roncero-Ramos et al., 2020), and microclimate (Belnap and Eldridge, 2001). Disturbance extent can influence the severity of these altered local conditions, for example, when landscape-level loss of vascular plants creates more connectivity at the soil surface and facilitates higher rates of sediment loss, which impedes natural recovery processes (Ravi et al., 2010). Thus, the reestablishment of biocrust organisms may also be dependent on the scale of the disturbance (Weber et al., 2016). Previous studies on biocrust natural recovery include a wide range of disturbance extents across studies, from 0.1 cm^2^ to 15 m^2^ (Belnap, (n.d.); Kidron et al., 2008; Dojani et al., 2011; Antoninka et al., 2018, 2020; Chock et al., 2019; Xiao et al., 2019), but few studies have assessed the impact of disturbance scale on biocrust natural recovery.

We evaluated the natural recovery of biocrusts after 1.5 years in response to small-scale disturbances ( ≤ 1 m^2^). While complete recovery of a biocrust community is expected to take much longer than 1.5 years, studies often start monitoring biocrust recovery within a few years after disturbance. To test how the spatial extent of physical disturbance impacts natural recovery, we scraped biocrust from plots of four different sizes at two sites on the Colorado Plateau. We hypothesized that the smallest disturbances (0.01 m^2^) would recover faster than larger disturbances (1 m^2^), assuming that lateral propagule dispersal would be enhanced for smaller disturbances and that smaller disturbances might promote more favorable conditions for natural recovery. This study is relevant to land managers who must consider restoration options for a variety of biocrust disturbances ranging from animal tracks to abandoned oil pads to miles of vehicle tracks. Being able to predict recovery rates based on disturbance extent may help guide soil and biocrust management decisions in drylands.

## 2. Materials and methods

### 2.1. Study sites

In October 2017, two sites were established in Canyonlands National Park Needles District in southeast Utah, USA: Greasewood Site (38° 9′6.17“N, 109°45′17.79”W) dominated by *Sarcobatus vermiculatus* and Sagebrush Site (38°11′6.10“N, 109°46′9.45”W) dominated by *Artemisia tridentata* (Table 1). The two sites are quite similar. Both sites are at ~1,500 m elevation and have an annual precipitation of 21.2 cm, an average annual maximum temperature of 20.3°C, and an average annual minimum temperature of 3.8°C (Western Regional Climate Center, (n.d.)). Soils at both sites belong to the Mido family (University of California Davis, 2022) and the Alkali Bottom (Greasewood) ecological site (USDA NRCS, 2022). Both sites have well-developed, rough biological soil crusts, co-dominated by moss (*Syntrichia* spp.) (Seppelt et al., 2016), lichen (*Collema* spp.) (Rosentreter et al., 2016), and cyanobacteria (*Microcoleus* spp.) (Anderson, (n.d.); Campbell et al., 2009). Canyonlands National Park was established in 1964, and the selected sites have experienced minimal disturbance since that time, though human use, including grazing, was likely a disturbance at the sites prior to the 1960's (Sheire, 1972). We did not expect the sites to experience significant physical disturbance throughout the experiment.

**Table 1:** The two sites were similar in climate, site stability, and disturbance factors and are thought to influence biological soil crust recovery (Belnap and Eldridge, 2001).

		**Greasewood site**	**Sagebrush site**
Climate	Location	Canyonlands National Park, Needles, USA	Canyonlands National Park, Needles, USA
	Elevation	~1,500 m	~1,500 m
	Precipitation (average annual)	21.2 cm	21.2 cm
	Temperature (average annual minimum and maximum)	3.8°C, 20.3°C	3.8°C, 20.3°C
Site stability	Soil texture	Sandy loam (6.5% clay)	Sandy loam (9% clay)
	Rock cover	Low rock cover	Low rock cover
	Shrub type and cover	Greasewood 15% cover	Big Basin Sagebrush 23% cover
	Biocrust cover	Pinnacled, rolling mature biocrusts	Pinnacled, rolling mature biocrusts
Disturbance	Intensity (may include fire, climate change, historic grazing, extreme erosional events with weather)	Low	Low
	Frequency	Rare	Rare

### 2.2. Experimental setup and sampling methods

In October 2017, we established disturbance treatment plots with the guidance of the National Park Service at each site with six replicates for each disturbance extent: 10 × 10 cm (100 cm^2^), 25 × 25 cm (625 cm^2^), 50 × 50 cm (2,500 cm^2^), and 100 × 100 cm (10,000 cm^2^). Throughout this article, we refer to these plots as “10,” “25,” “50,” and “100.” Replicates were positioned at least 3 m apart from one another, and each plot was at least 15 cm away from other plots (Supplementary Figure 1 depicts a representative site). Within each plot, biocrust was scraped off the soil surface down to 2 cm using a flat trowel, exposing the bare mineral soil underneath. Immediately after scraping the plots, biocrust cores (1 cm^3^, *n* = 5) were sampled in an “X” shape within the plots and evenly spaced, scaling with plot size, and then pooled. Cores were stored at −20°C for future analysis. We also established reference plots containing intact biocrusts (no disturbance) within 1 m of the disturbance treatment plots.

The plots were allowed to naturally recover for 1.5 years without intervention, except pin flags marking the corners of each plot left at the site. In May 2019, the plots were monitored for biocrust recovery by sampling each plot again at equally spaced locations along a transect through the midline of each plot. The “10” and “25” plots were sampled in three locations; the “50” and “100” plots were sampled at five locations. At each location, we sampled seven cores (1 cm^3^), which were pooled, air dried for 48 h, and then stored at 4°C until laboratory analysis. Soil stability (Slake) was assessed with a field-based soil aggregate stability test at each sample location (Herrick et al., 2001). Photographs of each plot were taken prior to sampling. A sampling scheme diagram is provided (Supplementary Figure 2).

As part of our assessment of site characteristics, we took five soil samples with a PVC ring and flat spatula (5.3 cm diameter, 5 cm depth) for soil texture analysis in the laboratory (Kettler et al., 2001). Next, we determined the shrub cover at each site with Google Earth Pro, 2016. A polygon (~1,500 m^2^) was used to delineate the sites, and then the area of each shrub was determined by drawing polygons to match the overhead area of each shrub within the site area. We calculated the percent cover as the total shrub area divided by the total site area, multiplied by 100. Finally, to assess temperature and precipitation for the recovery period as compared to long-term climate averages, we compared monthly average total precipitation and average temperature from October 2017 to May 2019 to the 95% confidence interval of the long-term mean for each month (1965–2022) (WRCC, Canyonlands Needles Station, https://wrcc.dri.edu/).

### 2.3. Laboratory methods

Potential photosynthetic activity is a common proxy for biocrust development and autotrophic organism biomass, quantified as the concentration of chlorophyll a in the soil (Ritchie, 2006; Castle et al., 2011). Higher soil chlorophyll a values indicate biocrust recovery toward reference levels of intact biocrusts. In this study, chlorophyll a was extracted from the soil following standardized procedures (Chock et al., 2019). Briefly, 1 g of homogenized soil (picked free of rocks and litter) was ground with 3 ml of 90% acetone for 3 min using a mortar and pestle. The mixture was transferred to a 15-ml Falcon tube using 10 ml of 90% acetone, vortexed for 2 min, and incubated at 4°C in the dark for 24 h. Samples were centrifuged (12 min, 4,000 rpm) and the supernatant separated for spectrophotometric measurement at 664 nm for chlorophyll a content and 750 nm for background adjustment (Ocean Optics, USB4000 with Ocean View Software, 2013, version 1.6.7). Chlorophyll a concentration was calculated as described in Ritchie (2006).

Soil exopolysaccharide (EPS) concentration provides an indicator of productivity, nutrient accumulation, and erosion resistance (Mallen-Cooper et al., 2020). We extracted EPS in three portions, namely, loosely bound EPS (L-EPS), tightly bound EPS (T-EPS), and glycocalyx EPS (G-EPS), which are defined by their extraction methods rather than by a characteristic composition or biological function; however, G-EPS is thought to make up cyanobacterial sheaths (Rossi et al., 2018). We calculate total EPS as the sum of all three EPS fractions; it represents all of the extractable extracellular polysaccharides in the soil. Soil exopolysaccharide content was determined with methods from Chock et al. (2019) with modifications to extract G-EPS, which is recommended for cyanobacterial-dominant biocrusts (Rossi et al., 2018) (e.g., early successional biocrusts). In brief, after extracting the L-EPS and T-EPS, the G-EPS was extracted by adding 500 μl of DI water to the previously extracted pellet, and the tube was heated to 80°C for 1 h (Rossi et al., 2018). The mixture was then centrifuged (5 min, 6,000 × g) and 200 μl of supernatant was separated off for the phenol sulfuric acid assay as described in Chock et al. (2019) using a plate reader with a monochromatic spectrophotometer (BioTek EL800, Winooski, VT) and a standard curve created from a stock D-glucose solution (200 mg/L). The EPS concentration of each sample was calculated using a calibration curve and the dry weight of each sample. Total EPS was calculated as the sum of each fraction (LB-EPS + TB-EPS + G-EPS) for a given sample.

### 2.4. Statistical analyses

We used linear models to test for biocrust's natural recovery. For each model, we checked the assumptions for equal variance and a normal distribution of the residuals. To meet these requirements, we used either a log or square root transformation, as necessary. The site was excluded from all models after model selection via an analysis of variance between models with and without the site as a random effect. For all tests, we used a 95% significance level. We assessed the recovery of biocrust chlorophyll a and EPS content as compared to the freshly disturbed soils with the following predictor levels: scraped, “10,” “25,” “50,” or “100.” We used Helmerts contrasts for each level, which is a method for comparing each level against the average of the higher levels successively (Crawley, 2007). Next, we found median soil stability at the plot level from replicate measurements and used Fisher's exact test to determine the relationship between median soil stability and disturbance extent (Siegel, 1957; McCrum-Gardner, 2008). All analyses were conducted in R (4.0.2) with RStudio (1.3.1093).

## 3. Results

**Figure 1:**
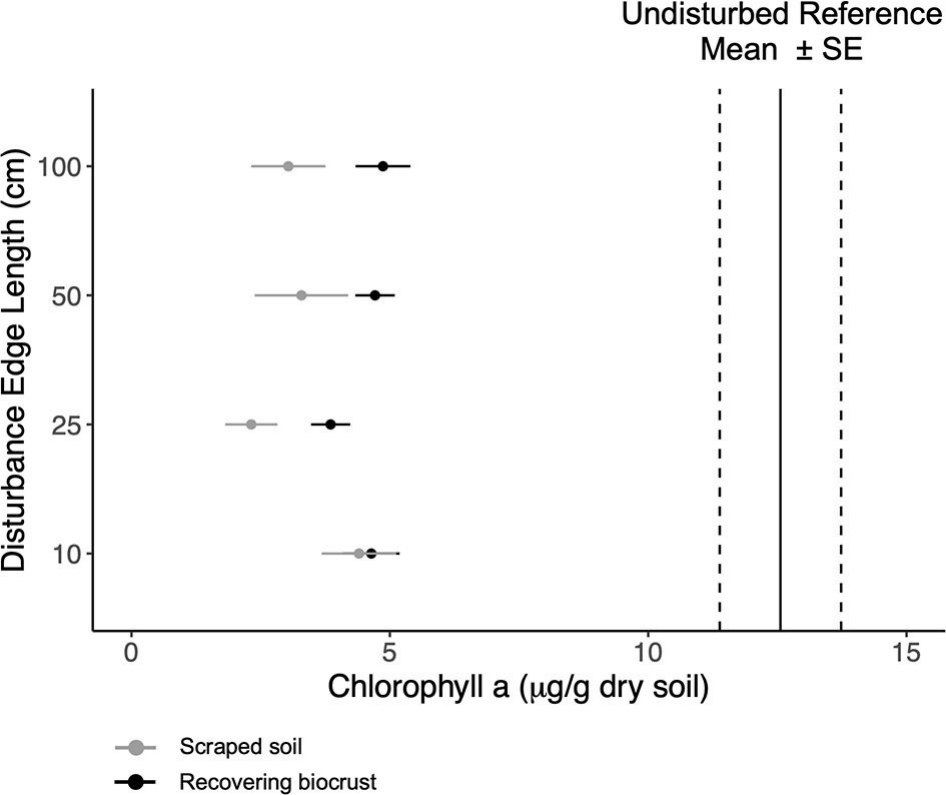
Mean chlorophyll a content (points) and standard error for the scraped soils (gray) and the recovering biocrusts (black). Mean chlorophyll a content (solid black lines) with standard error (dashed lines) for reference biocrusts. The disturbance scale increases up the y-axis.

To assess biocrust recovery 1.5 years after the disturbance treatment, we measured chlorophyll a and exopolysaccharide (EPS) content, which are proxies for biocrust biomass and productivity. We also measured soil aggregate stability, a proxy for erosion resistance. Chlorophyll a concentration did not differ by disturbance scale (Figure 1 and Supplementary Table 4). On average, chlorophyll a increased 1.5-fold in 1.5 years, increasing from ~2.7 μg g^−1^ dry soil after scraping the soil to ~4.3 μg g^−1^ dry soil. Target chlorophyll a concentration was ~12–13 (± 2) μg g^−1^ dry soil for the neighboring reference biocrusts (Supplementary Table 1).

**Figure 2:**
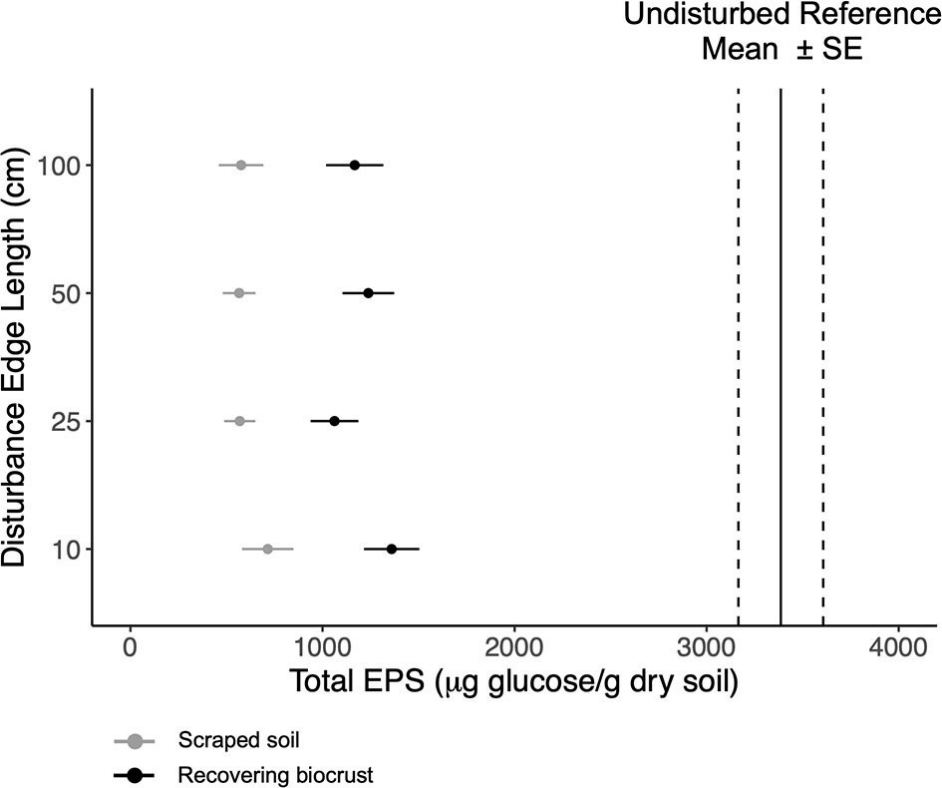
Mean total EPS content (points) with standard error for the scraped soils (gray) and the recovering biocrusts (black). Mean total EPS content (solid black lines) with standard error (dashed lines) for reference biocrusts. The disturbance scale increases up the y-axis.

Similarly, total EPS concentrations were not affected by the disturbance scale (Figure 2). On average, total EPS doubled in concentration in 1.5 years, increasing from ~560 μg glucose g^−1^ dry soil after scraping the soil to ~1,126 μg glucose g^−1^ dry soil. Target total EPS concentrations were ~3,000–3,700 (±200) μg glucose g^−1^ dry soil (Supplementary Table 2). Loosely bound and tightly bound EPS followed the same pattern as total EPS. However, the smallest plots had higher glycocalyx EPS as compared to larger disturbance extents (linear model, *t* = 2.2, *p*-value = 0.035). The smallest disturbance extent contained an average of ~550 ± 50 μg glucose per cm^3^ compared to an average of ~420 ± 50 μg glucose per cm^3^ in larger disturbance extents. For detailed information about each EPS fraction, see Supplementary Table 4.

**Figure 3:**
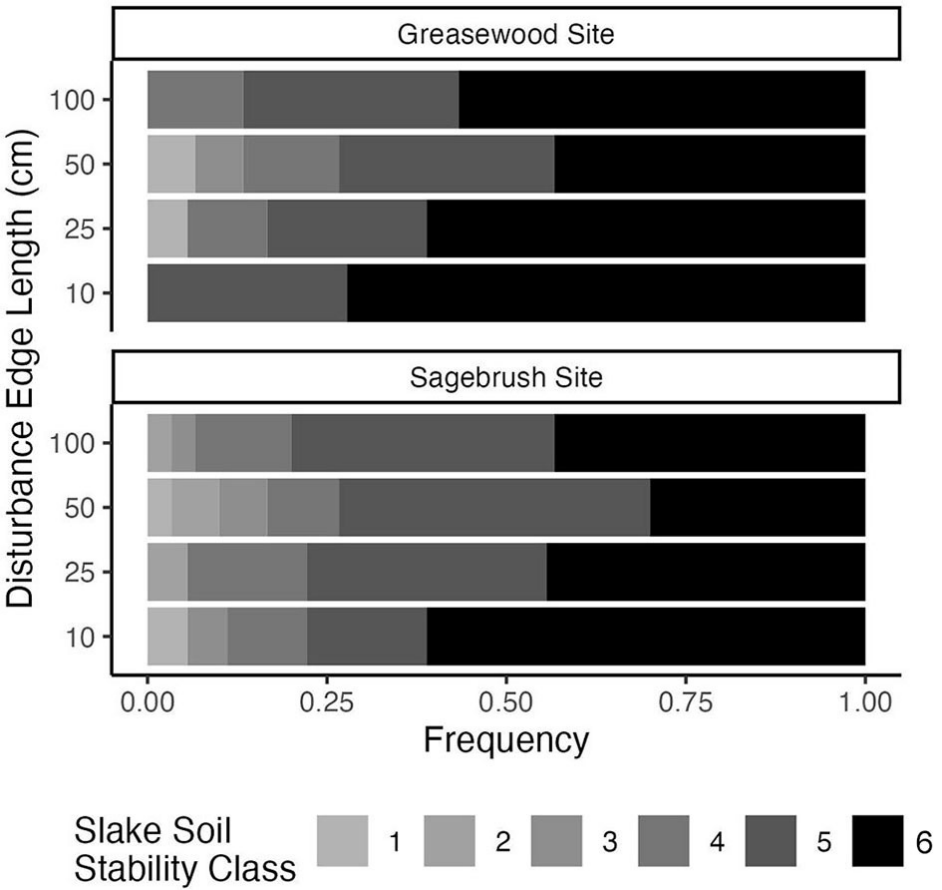
Soil stability class frequencies for each disturbance scale at two sites (*n* = 6). The darker colors represent higher soil stability on the Slake scale of 1–6, while lighter colors represent lower soil stability.

Recovering biocrust soils reached high slake stability levels but did not significantly differ by disturbance scale. However, we found a trend in soil stability recovery across disturbance extents that held across sites. Though not statistically significant, the highest soil stability class was observed more frequently in the smallest plots; soil stability decreased as disturbance extent increased, and finally, soil stability increased slightly for the largest plot size (Figure 3). Soil stability differed slightly by the site (Fisher's exact test, *p* = 0.035) with a median of 6 (median absolute deviation = 0) at the Greasewood Site and a median of 5 (median absolute deviation = 0) at the Sagebrush Site.

### 3.1. Context of biocrust recovery

**Figure 4:**
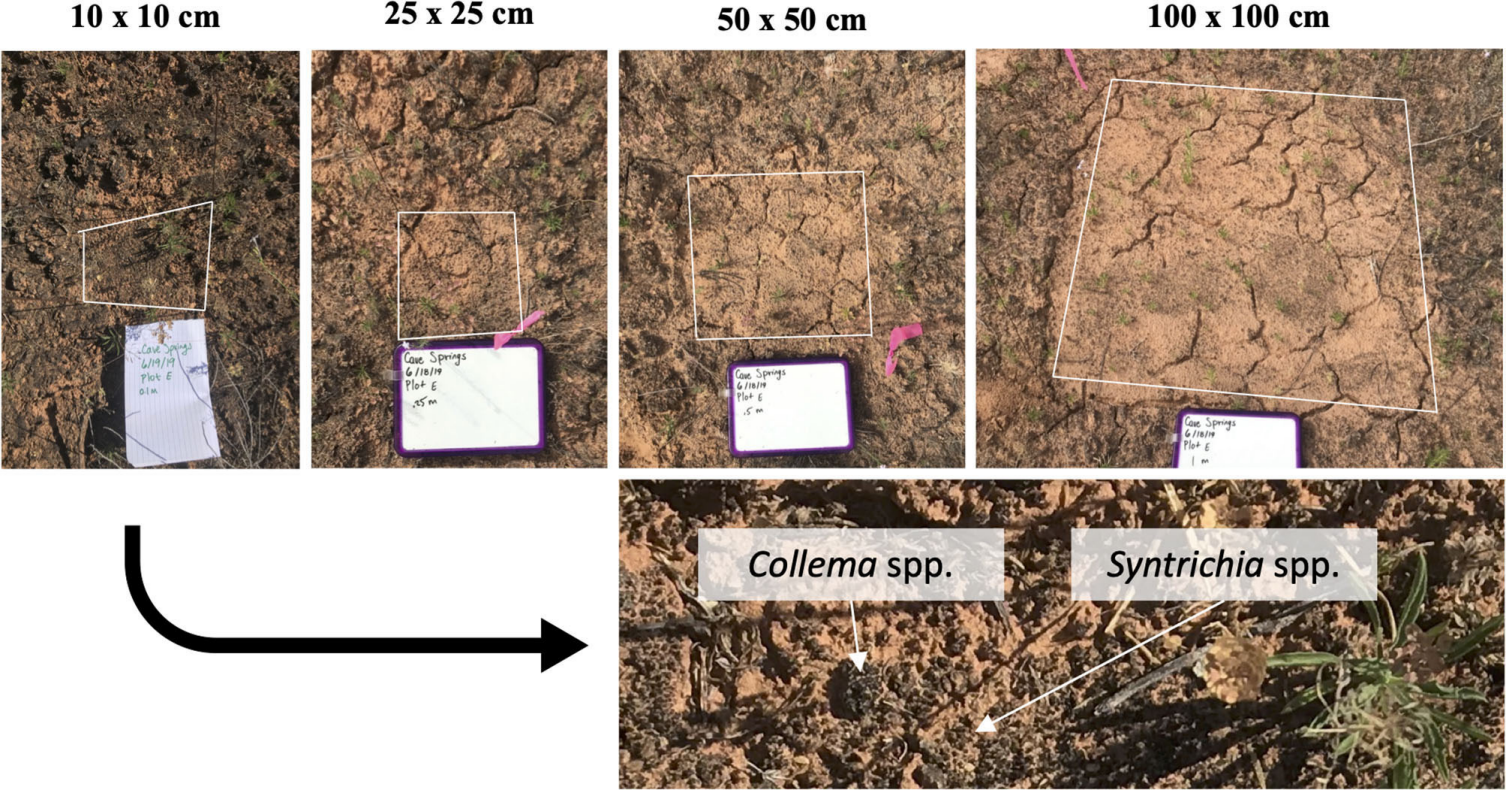
One set of disturbance extents from the Greasewood Site on the Colorado Plateau 1.5 years after scraping disturbance. The darkening of the soil surface indicates the colonization and growth of cyanobacteria, moss, and lichen. Variability in colonization can be observed in the “100” plots (top right), with dark patches of growth in the lower half of the disturbance area. Lichen (*Collema* spp.) and moss (*Syntrichia* spp.) were important colonizers of all plots in addition to cyanobacteria.

After 1.5 years of natural recovery, biocrust growth was visible in all treatment plots at both sites (Figure 4 and Supplementary Figures 3, 4), including mosses (*Syntrichia* spp.), lichens (*Collema* spp.), and filamentous cyanobacteria. Biocrust colonization of disturbed plots ranged widely; for some plots, moss and lichen colonization was visibly homogenous throughout the disturbed area, and in other cases, growth was patchy.

The monthly average temperature and monthly average precipitation during the period of biocrust recovery were typical for the area. In the first year of recovery (Oct 2017–Oct 2018), the area received 21.3 cm of precipitation, and the next 7-month period prior to monitoring received 15.9 cm of precipitation. During the recovery period, 2 months experienced above-average precipitation: October 2018 and February 2019, as determined by comparing monthly precipitation totals to the 95% confidence interval for the long-term average total precipitation (1965–2022) (WRCC, Canyonlands Needles Station, https://wrcc.dri.edu/). Monthly average temperatures throughout the recovery period fell within the 95% confidence intervals for the long-term average temperature (1965–2022) (WRCC, Canyonlands Needles Station, https://wrcc.dri.edu/).

Our disturbance treatment was effective at removing biocrust organisms as compared to intact biocrust communities surrounding the disturbances. The scraping disturbance reduced chlorophyll a concentration by 75%, total EPS by 82%, and all three EPS fractions by 78–87% as compared to the intact reference biocrusts. Soil stability was substantially impacted by the disturbance treatment but was not measured in the field in 2017.

## 4. Discussion

In this study, we present conflicting evidence for whether disturbance extent influences biocrust natural recovery rates. We found that chlorophyll a, total EPS, loosely bound EPS, and tightly bound EPS do not recover differently due to disturbance extent; however, we found that glycocalyx EPS recovered to higher concentrations in small disturbances. Soil stability also showed trends toward higher stability in smaller plots. Therefore, some of our results support our initial hypothesis that smaller disturbances recover faster. This may be due to sub-plot variability in recovery coupled with the way samples were pooled at the plot level prior to analysis or perhaps this could be due to differences in the contribution of different biocrust organisms to the various metrics and their consistency over space or time. For instance, total EPS may capture the net EPS production and degradation for the whole biocrust community, while G-EPS concentrations may be more closely correlated to cyanobacteria productivity since they are associated with the cyanobacteria sheath (Rossi et al., 2018). It has previously been shown that G-EPS has a higher correlation with chlorophyll a than other EPS fractions (see Chock et al., 2019), suggesting a stronger connection between G-EPS and cyanobacteria productivity.

The experimental disturbance treatments in this study were surrounded by intact mature biological soil crust communities, thus we initially expected the primary source of propagules to the newly disturbed plots would be from lateral movement. Based on estimates of cyanobacterial expansion in the laboratory (Sorochkina et al., 2018), cyanobacteria could fully colonize the “10,” “25,” and “50” plots within a 1.5-year timeframe under ideal conditions. Alternatively, the “100” plots would not be fully colonized within this time frame under laboratory conditions. However, lateral expansion alone does not fully reflect the observed recovery pattern of biocrust. If lateral growth was primarily responsible for biocrust reestablishment, we would expect to see a more regular pattern of biocrust succession (Read et al., 2016; Elliott et al., 2019; Roncero-Ramos et al., 2020) inward from the plot edges, potentially with more developed crusts at the edges. Instead, it is likely that wind or water strongly influenced the observed recovery patterns and created high sub-plot variability. Our sampling strategy of pooling samples across the plot means that areas of high recovery were diluted by areas of no recovery at the plot level, which may have a larger impact on measurements associated with larger disturbance extents. The importance of wind and water for recolonization also suggests that propagules may originate from both intact communities in the immediate vicinity of the disturbance and from a much larger area if transported via wind or water. This may be especially true in this study, where intact biocrusts exist within and around Canyonlands National Park, which is protected from most ground-disturbing activities.

The two sites selected for this study differed primarily in shrub type. Shrubs can influence microsite characteristics at the soil surface, such as soil moisture and water movement, litter inputs, and wind patterns (Bochet et al., 2006; Dettweiler-Robinson et al., 2013). Site differences did not play an important role in biocrust's natural recovery in this study. The only site difference we found was soil stability, with the Greasewood Site recovering to higher soil stability than the Sagebrush Site, though disturbed biocrusts at both sites recovered to high stability levels within just 1.5 years. While there were slight differences in the sites in terms of shrub type, shrub cover, and soil texture, these differences were not enough to significantly impact the measured chlorophyll a or EPS content, as has been shown for other studies (Weber et al., 2016; Chock et al., 2019; Faist et al., 2020).

**Figure 5:**
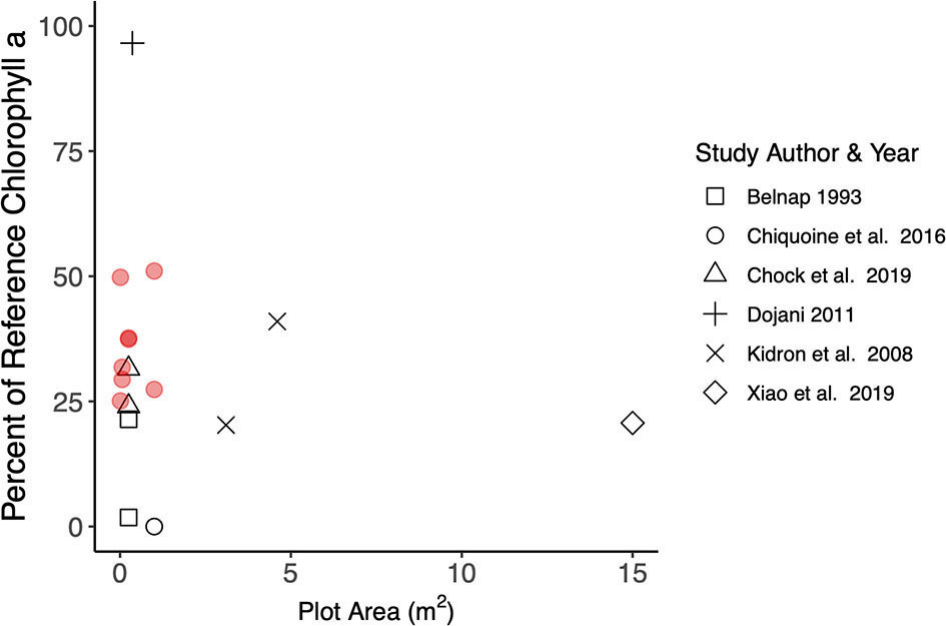
Mean chlorophyll a content for recovering biocrusts as a percentage of reference biocrust chlorophyll a reported in each study. Studies were included that measured biocrust recovery 2 years after a scraping disturbance and when disturbance scales were reported. The mean values from this study are shown in red circles, while those from other studies are shown in black. There were no trends between disturbance scale and percent recovery of chlorophyll a in the first few years after disturbance.

The recovery we measured for two sites on the Colorado Plateau (i.e., high soil stability after 1.5 years and moderate recovery of chlorophyll a and total EPS concentrations) is in line with previous measures of biocrust natural recovery in other drylands. In addition, we can compare our study to other studies that use control plots for experimental biocrust inoculation since they are sufficiently similar to biocrust natural reestablishment. Across all plots, our study found an annual increase of 1 μg chlorophyll a g^−1^ year^−1^ and median soil stability of 5.5, which is similar to 1 μg chlorophyll a g^−1^ year^−1^ with median soil stability of 5 (Chock et al., 2019), to 3 μg g^−1^ year^−1^ with median soil stability of 4 (Faist et al., 2020), and to 2.5 μg g^−1^ year^−1^ with median soil stability of 5 (Antoninka et al., 2020). Several studies have used a variety of biocrust metrics (e.g., moss stem density and NDVI (Zaady et al., 2007), protein content (Kidron et al., 2008), and species composition or cover (Belnap, (n.d.); Langhans et al., 2010) to understand biocrust natural recovery following scraping disturbances. Different metrics for biocrust indicate different characteristics of recovery, and it can be challenging to link these recovery metrics to one another, especially when different methods are used or values are reported differently across studies. Regardless, biocrust reestablishment studies from drylands tend to agree that natural recovery of biological soil crust can begin within a short timeframe following a severe disturbance, but full recovery to target levels of development, diversity, and function takes much longer (Weber et al., 2016). Furthermore, the rate of recovery may differ based on the metric used. For instance, soil stability tended to increase dramatically in just a few years after disturbance, while measures of productivity (e.g., chlorophyll a) may take much longer or be more variable over time (Weber et al., 2016). Finally, across biocrust natural recovery studies, there does not appear to be an effect of disturbance extent on chlorophyll a (Figure 5), and few studies include disturbances larger than 1 m^2^.

Our study indicates that biocrust disturbances < 1 m^2^ in size may be expected to recover at similar rates if other factors, like climate and site characteristics, are consistent, though recolonization should not be expected to occur consistently in space. Our results are relevant to land managers making decisions about supporting biocrust natural recovery vs. employing active restoration strategies. We encourage future research to assess larger disturbance extents relevant to land management (e.g., an oil pad or large-scale grazing disturbance). Propagule dispersal limitation, disturbance characteristics, and site characteristics may be substantially different at scales larger than 1 m^2^, so assessments of biocrust recovery for large disturbance extents are important for decision-making in land management and for prioritizing restoration actions. The largest experimental studies of severe biocrust disturbance (i.e., scraping) have been conducted on 15-m^2^ plots (Xiao et al., 2019), and the majority of studies are conducted on plots < 1 m^2^. While these studies are important for understanding mechanisms of dispersal and succession, they do not capture larger scale patterns and processes that are also relevant to the current and future disturbance landscape in drylands. We also note that our study only addressed the disturbance scale for the complete removal of biocrust, but there are many other important disturbance types (e.g., fire or chemical treatments), which are less commonly studied, that could assess the importance of disturbance extent on biocrust recovery. Monitoring biocrust recovery surrounding large development projects, utilizing control sites for biocrust restoration projects, or developing space-for-time analyses of previous disturbances may offer opportunities for such a study.

## Data availability statement

The raw data supporting the conclusions of this article will be made available by the authors, without undue reservation.

## Author contributions

CH and NB conceptualized the experiment. CH obtained permits and installed the field experiment. SJ completed field monitoring, soil chemistry, data analysis, and the preparation of the first version of the manuscript. SJ, CH, and NB contributed equally to the final version of the manuscript. All authors contributed to the article and approved the submitted version.
