# Supplementary material for: The influence of disturbance scale on the natural recovery of biological soil crusts on the Colorado Plateau
Source: Front Microbiol. 2023 Aug 3;14:1176760. doi: 10.3389/fmicb.2023.1176760 (PMC10434622; doi:10.3389/fmicb.2023.1176760)
Supplement: Supplementary file 1 [file Data_Sheet_1.pdf]

Supplementary Material

The influence of disturbance scale on the natural recovery of biological soil crusts on the Colorado Plateau

Sierra D. Jech*, Caroline A. Havrilla, Nichole N. Barger

*** Correspondence:** Corresponding Author: sierra.jech@colorado.edu

1 Supplementary Figures and Tables

1.1 Supplementary Figures

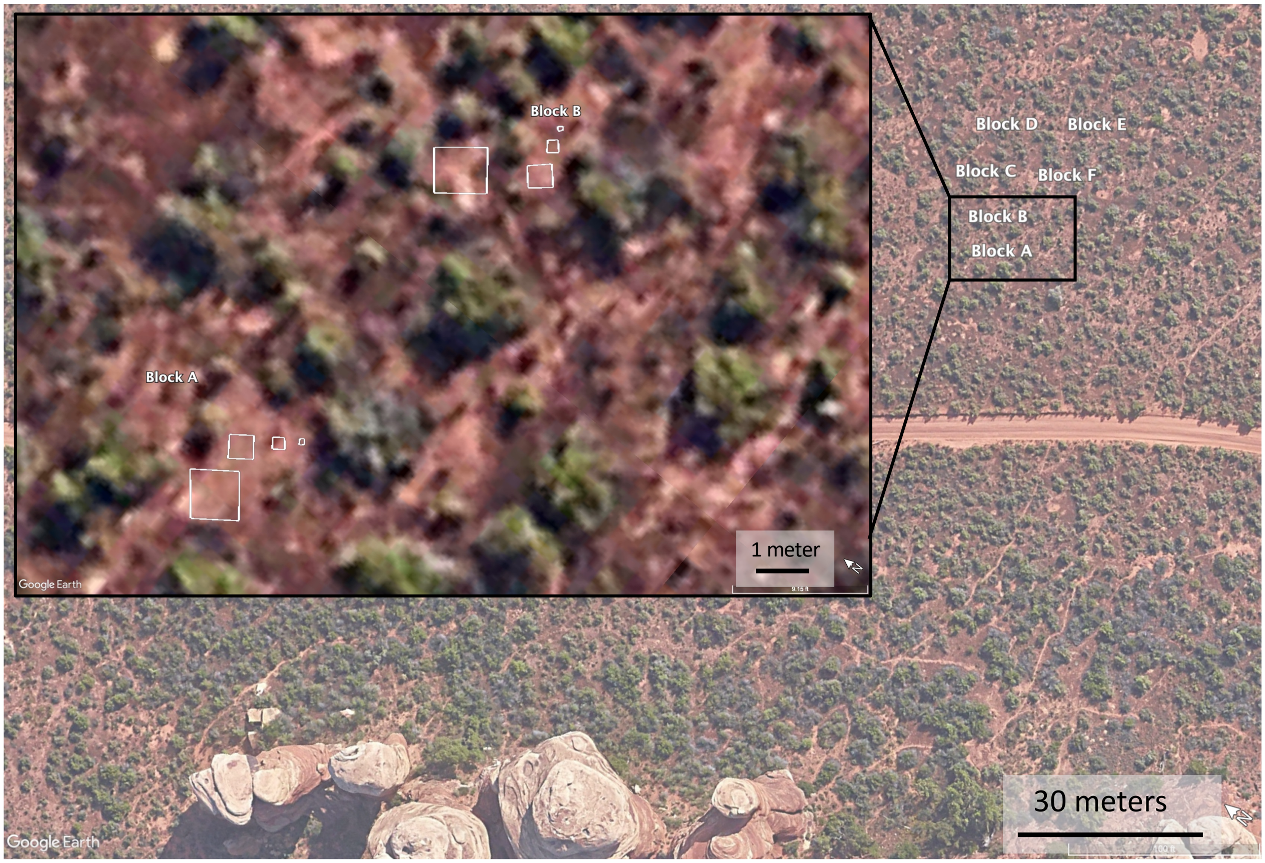

Supplementary Figure 1. Map of the sagebrush site with six replicate blocks depicted. The inset shows a more detailed view of the site and representative disturbance extents for two replicates. Imagery from Google Earth Pro.

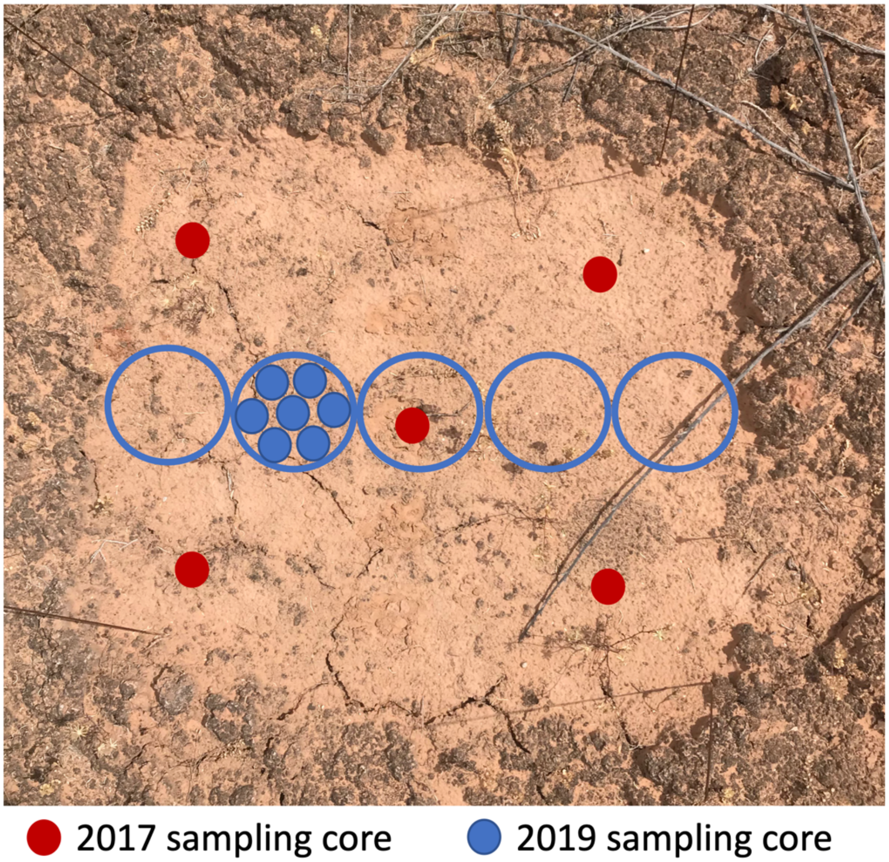

Supplementary Figure 2. Sampling scheme immediately after the scraping disturbance (red 2017 points) and 1.5 years later (blue 2019 points).

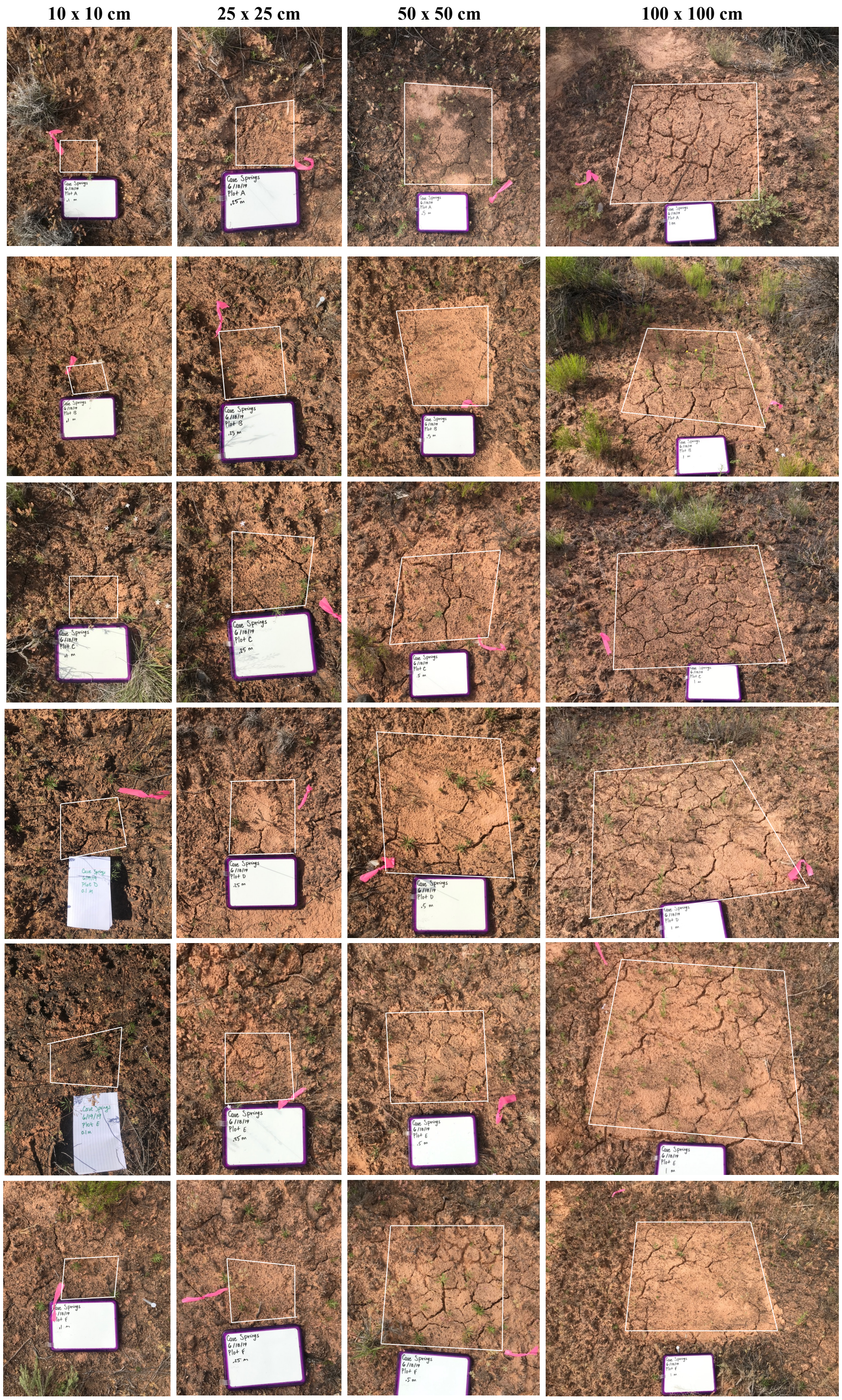

Supplementary Figure 3. Images show all six replicates of the disturbed plots at the Greasewood Site after 1.5 years of recovery and the neighboring intact biological soil crusts for comparison. Each disturbance scale (10, 25, 50, and 100) is shown in the four columns (left to right).

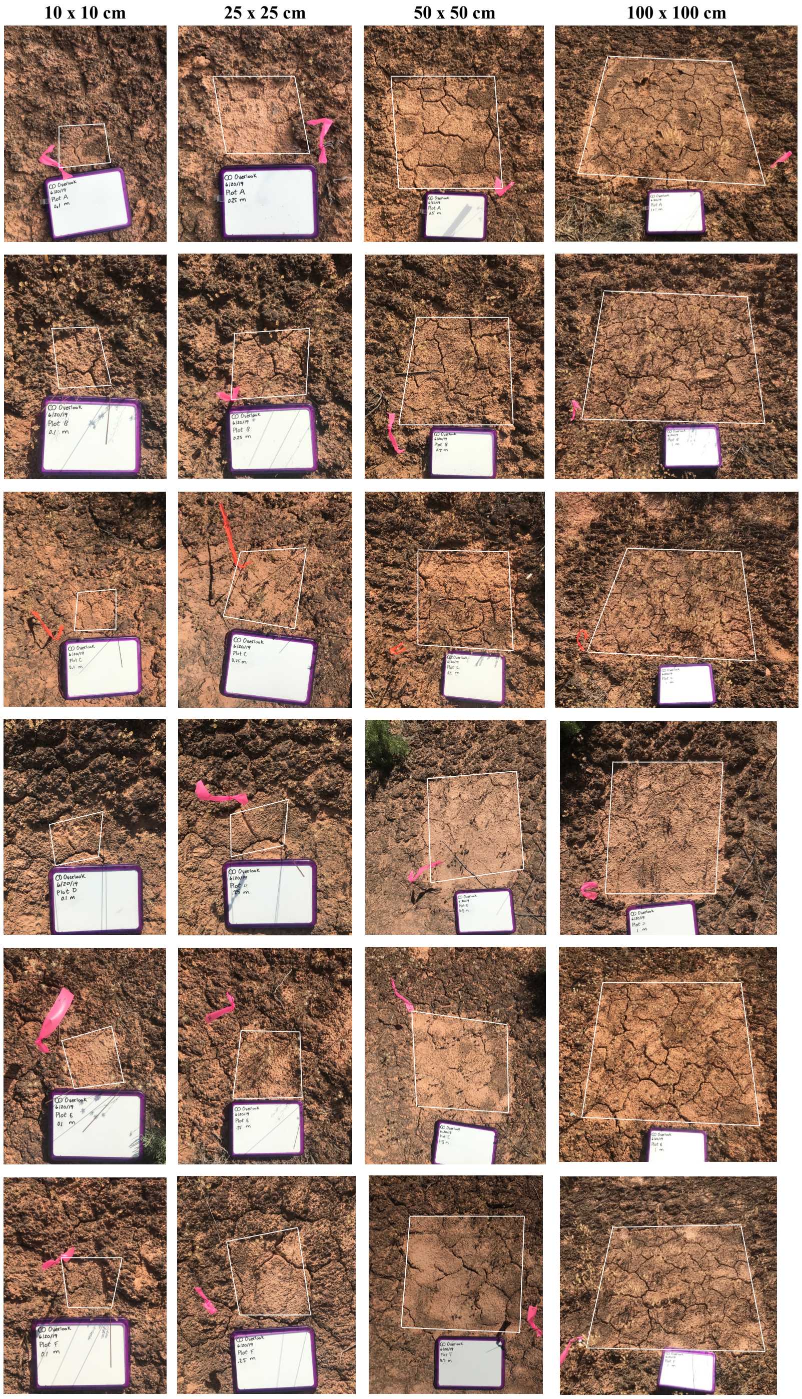

Supplementary Figure 4. Images show all six replicates of the disturbed plots at the Sagebrush Site after 1.5 years of recovery and the neighboring intact biological soil crusts for comparison. Each disturbance scale (10, 25, 50, and 100) is shown in the four columns (left to right).

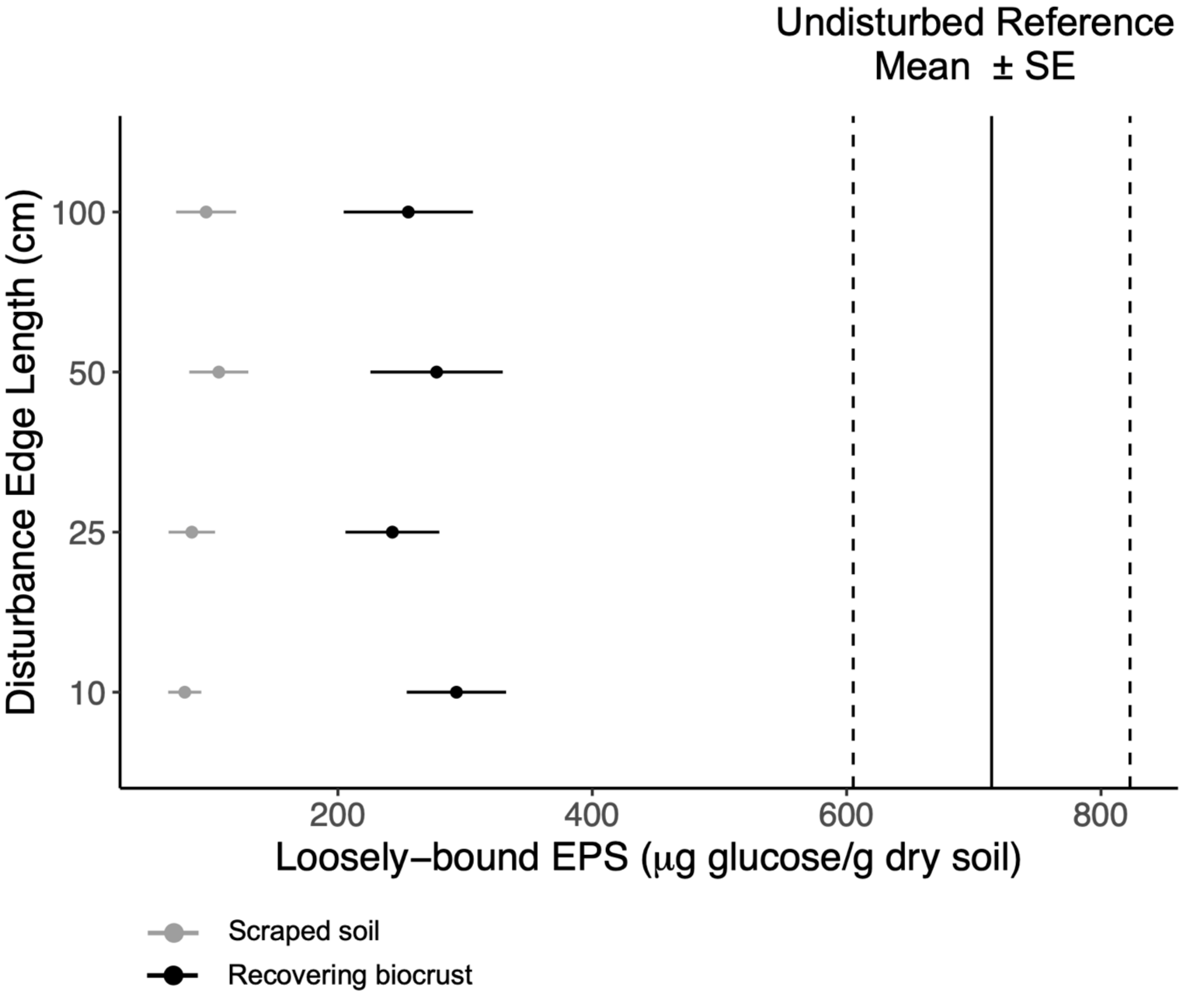

Supplementary Figure 5. Mean loosely bound EPS content (points) and standard error for the scraped soils (grey) and the recovering biocrusts (black). Mean loosely bound EPS content (solid black lines) with standard error (dashed lines) for reference biocrusts. Disturbance scale increases up the y axis.

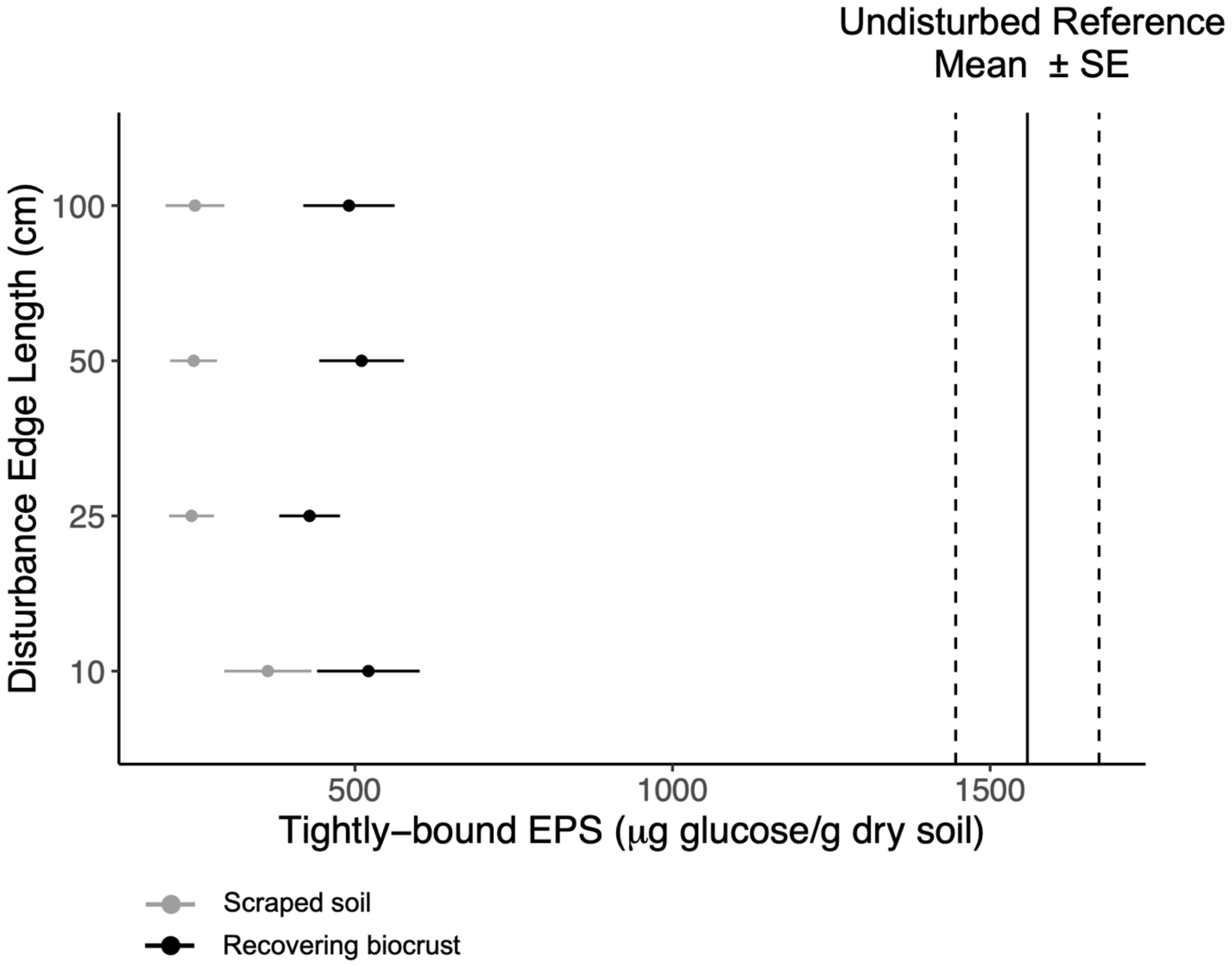

Supplementary Figure 6. Mean tightly bound EPS content (points) and standard error for the scraped soils (grey) and the recovering biocrusts (black). Mean tightly bound EPS content (solid black lines) with standard error (dashed lines) for reference biocrusts. Disturbance scale increases up the y axis.

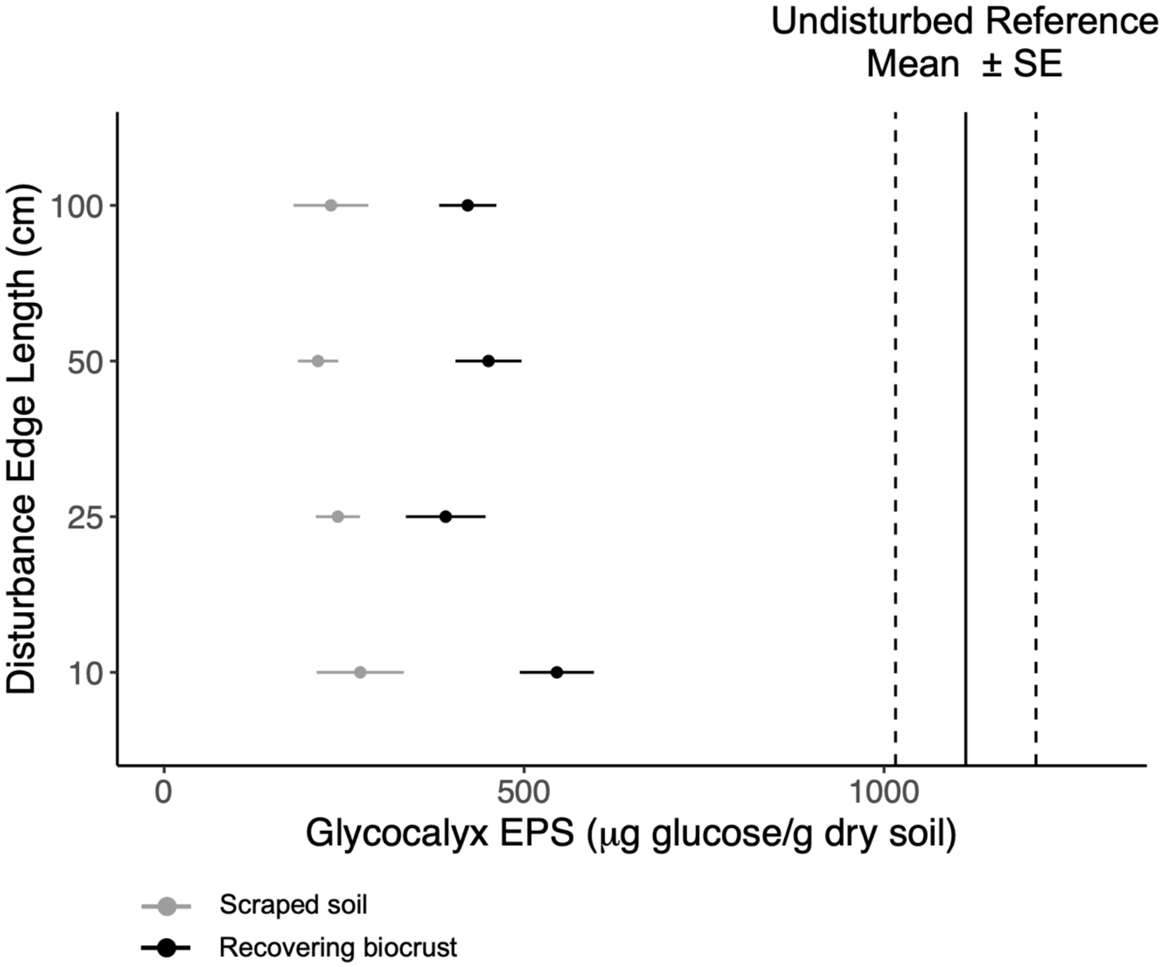

Supplementary Figure 7. Mean glycocalyx EPS content (points) and standard error (error bars) for the scraped soils (grey) and the recovering biocrusts (black). Mean glycocalyx EPS content (solid black lines) with standard error (dashed lines) for reference biocrusts. Disturbance scale increases up the y axis.

1.2 Supplementary Tables

Supplementary Table 1. Mean chlorophyll a concentration ± SE (μg/cm^3) for scraped, recovering, and target biocrusts at each disturbance scale.

Disturbance Edge (cm)	Scraped chlorophyll a mean ± se	Recovering chlorophyll a mean ± se	Target chlorophyll a mean ± se
10	4.4 ± 0.7	4.6 ± 0.6	12.6 ± 1.2
25	2.3 ± 0.5	3.9 ± 0.4	
50	3.3 ± 0.9	4.7 ± 0.4	
100	3.0 ± 0.7	4.9 ± 0.5	

Supplementary Table 2. Mean EPS concentration ± SE (µg/cm^3) for scraped, recovering, and target biocrusts for each disturbance scale. Loosely bound EPS, tightly bound EPS, glycocalyx EPS, and total EPS values provided.

	**Disturbance Edge (cm)**	**Scraped EPS mean ± se**	**Recovering EPS mean ± se**	**Target EPS mean ± se**
Loosely bound EPS	10	80 ± 13	293 ± 39	714 ± 109
	25	85 ± 18	243 ± 37	
	50	106 ± 23	278 ± 52	
	100	96 ± 24	255 ± 51	
Tightly bound EPS	10	363 ± 69	521 ± 81	1559 ± 113
	25	243 ± 35	429 ± 48	
	50	246 ± 37	510 ± 67	
	100	248 ± 46	491 ± 72	
Glycocalyx EPS	10	272 ± 61	546 ± 52	1114 ± 98
	25	241 ± 31	391 ± 55	
	50	214 ± 28	451 ± 46	
	100	232 ± 52	422 ± 40	
Total EPS	10	715 ± 134	1360 ± 144	3386 ± 221
	25	569 ± 81	1062 ± 124	
	50	566 ± 85	1239 ± 134	
	100	576 ± 116	1168 ± 149	

Supplementary Table 3. Linear models comparing freshly disturbed soils to the reference biocrusts for chlorophyll a, total EPS, loosely bound EPS, tightly bound EPS, and glycocalyx EPS. The chlorophyll a and glycocalyx EPS models exclude the random effect of site because the variance due to site was zero. The other models exclude the random effect of site based on model comparisons (ANOVA comparison with and without the site random effect are included in the table). Comparison of means, standard error, t-value, and p-value for the categorical fixed effect are reported as transformed values. Significant difference in mean is shown in bold.

	**Mean**	**Std. Error**	**t value**	**p value**
square root (chlorophyll a) ~ disturbance extent				
**Intact - Disturbed**	**-1.8**	**0.24**	**-7.46**	**4.02e-08**
Site excluded because no model variance could be attributed to site.				
square root (total EPS) ~ disturbance extent				
**Intact – Disturbed**	**-33.90**	**2.29**	**-14.81**	**8.93e-15**
Site excluded as a random effect based on model comparison (ANOVA, X^2 = 0.475, p = 0.491)				
log (lightly bound EPS) ~ disturbance extent				
**Intact – Disturbed**	**-2.10**	**0.22**	**-9.59**	**2.41e-10**
Site excluded as a random effect based on model comparison (ANOVA, X^2 = 0.185, p = 0.667)				
square root (tightly bound EPS) ~ disturbance extent				
**Intact – Disturbed**	**-23.12**	**1.62**	**-14.26**	**2.31e-14**
Site excluded as a random effect based on model comparison (ANOVA, X^2 = 0.533, p = 0.466)				
square root (glycocalyx EPS) ~ disturbance extent				
**Intact – Disturbed**	**-18.09**	**1.58**	**-11.47**	**4.29e-12**
Site excluded because no model variance could be attributed to site.				

Supplementary Table 4. Linear models with helmerts contrasts comparing freshly disturbed soils to the recovering biocrusts. The models exclude the random effect of site based on model comparisons (ANOVA comparison with and without the site random effect are included in the table). The model estimate, t value, and p-value are given for the disturbance extent predictor as compared to higher contrast levels, reported as transformed values when applicable. Significant comparisons are bold.

	**Estimate**	**Std. Error**	**t value**	**p value**
chlorophyll a ~ disturbance extent				
**Intercept**	**2.02**	**0.05**	**37.54**	**< 2e-16**
**Scrape – Recovering 10, 25, 50, 100**	**-0.37**	**0.11**	**-3.38**	**0.001**
Recovering 10 – Recovering 25, 50, 100	0.10	0.15	0.67	0.504
Recovering 25 – Recovering 50, 100	-0.25	0.16	-1.60	0.115
Recovering 50 – Recovering 100	-0.04	0.18	-0.21	0.833
Site excluded because no model variance could be attributed to site.				
log (total EPS) ~ disturbance extent				
**Intercept**	**6.89**	**0.05**	**147.79**	**< 2e-16**
**Scrape – Recovering 10, 25, 50, 100**	**-0.70**	**0.10**	**-7.40**	**2.89e-10**
Recovering 10 – Recovering 25, 50, 100	0.17	0.13	1.36	0.178
Recovering 25 – Recovering 50, 100	-0.14	0.13	-1.00	0.319
Recovering 50 – Recovering 100	0.06	0.16	0.36	0.718
Site excluded as a random effect based on model comparison (ANOVA, X^2 = 0.470, p = 0.493)				
log (lightly bound EPS) ~ disturbance extent				
**Intercept**	**5.22**	**0.07**	**76.26**	**< 2e-16**
**Scrape – Recovering 10, 25, 50, 100**	**-1.03**	**0.14**	**-7.35**	**3.59e-10**
Recovering 10 – Recovering 25, 50, 100	0.20	0.19	1.05	0.300
Recovering 25 – Recovering 50, 100	-0.02	0.20	-0.08	0.939
Recovering 50 – Recovering 100	0.12	0.23	0.54	0.591
Site excluded because no model variance could be attributed to site.				

square root (tightly bound EPS) ~ disturbance extent				
**Intercept**	**20.47**	**0.56**	**36.45**	**< 2e-16**
**Scrape – Recovering 10, 25, 50, 100**	**-5.32**	**1.15**	**-4.64**	**1.67e-05**
Recovering 10 – Recovering 25, 50, 100	0.77	1.53	0.50	0.616
Recovering 25 – Recovering 50, 100	-1.55	1.62	-0.96	0.343
Recovering 50 – Recovering 100	0.32	1.87	0.17	0.865
Site excluded as a random effect based on model comparison (ANOVA, $X^2 = 1.09$, p = 0.296)				
log (glycocalyx EPS) ~ disturbance extent				
**Intercept**	**5.91**	**0.05**	**119.97**	**< 2e-16**
**Scrape – Recovering 10, 25, 50, 100**	**-0.66**	**0.10**	**-6.60**	**7.89e-09**
**Recovering 10 – Recovering 25, 50, 100**	**0.29**	**0.13**	**2.16**	**0.035**
Recovering 25 – Recovering 50, 100	-0.19	0.14	-1.36	0.180
Recovering 50 – Recovering 100	0.035	0.16	0.21	0.833
Site excluded as a random effect based on model comparison (ANOVA, $X^2 = 0.511$, p = 0.474)				

Supplementary Table 5. T-test comparing average values for recovering biocrusts to average intact reference biocrusts. Significant comparisons are bold.

	t	df	p-value
**Chlorophyll a**	**-6.70**	**5.4**	**8.1e-4**
**Total EPS**	**-9.42**	**6.0**	**8.03e-05**
**Lightly bound EPS**	**-4.02**	**5.4**	**8.6e-3**
**Tightly bound EPS**	**-9.10**	**5.9**	**1.1e-4**
**Glycocalyx EPS**	**-6.57**	**5.7**	**7.5e-4**
